# Influence of Carbon Sorbent Quantity on Breakthrough Time in Absorbent Filters for Antismog Half Mask Application

**DOI:** 10.3390/ma15020584

**Published:** 2022-01-13

**Authors:** Agnieszka Brochocka, Aleksandra Nowak, Paweł Kozikowski

**Affiliations:** 1 Department of Personal Protective Equipment, Central Institute for Labour Protection-National Research Institute, 90-133 Lodz, Poland; alnow@ciop.lodz.pl; 2 Department of Chemical, Biological and Aerosol Hazards, Central Institute for Labour Protection-National Research Institute, 00-701 Warsaw, Poland; pakoz@ciop.pl

**Keywords:** antismog half mask, carbon sorbent, breakthrough time, textural parameters

## Abstract

In this article, we present polymer non-woven fabrics with the addition of carbon sorbents being tested to estimate the breakthrough time and efficient protection against vapors present in smog. For this purpose, three substances were selected, which constitute an inhalation hazard and are smog components: cyclohexane, toluene, and sulfur dioxide. It was demonstrated that an increased quantity of carbon sorbent in polymeric filters significantly prolongs the breakthrough time. However, high sorbent quantities may increase the filter surface mass and air flow resistance. To optimize the protective parameters with functionality, a compromise between the two has to be found. By comparing the breakthrough times for different carbon sorbent quantities, the optimal filter composition was elaborated. The analyzed non-woven fabrics were manufactured by the melt-blown process and filled with ball-milled carbon sorbents supplied directly into the fabric blowing nozzle. Both protective performance and textural properties were analyzed for two commercially available carbon sorbents. Furthermore, it was proven that high values of sorbent-specific surface area translates directly into greater filter performance.

## 1. Introduction

In the first half of the XX century, several air pollution episodes occurred, with dramatic immediate effects on mortality: Meuse valley, Belgium, in 1930; Donora, PA, USA in 1948; and the most dramatic of all, London, UK, 1952. These episodes were all caused by a combination of high local pollution emissions and stagnant weather conditions preventing the atmospheric dispersal of pollution. The London 1952 episode resulted in 4000 additional deaths in the first week, and 8000 more in the following 2 months [1].

Recent studies show a direct correlation between long-term exposure to air pollution and increased risk of developing diseases of the respiratory tract, such as Influenza or even COVID-19 [2,3,4,5]. The study, published in the scientific journal *Cardiovascular Research Today*, estimated that about 15% of deaths worldwide from COVID-19 could be attributed to long-term exposure to air pollution [3]. In Europe, the proportion was about 19%, in North America it was 17%, and in East Asia about 27%. In their CVR paper, the researchers wrote that these proportions are an estimation of “the fraction of COVID-19 deaths that could be avoided if the population were exposed to lower counterfactual air pollution levels without fossil fuel-related and other anthropogenic (caused by humans) emissions” [3].

Today, numerous studies confirm the negative impact of air pollution on the physical and chemical properties of the natural atmosphere [6,7,8]. The findings of most studies demonstrate that both short- and long-term exposure to air pollution, especially PM 2.5 and, to a lesser extent, PM 10, sulfur dioxide (SO_2_) and nitrogen dioxide (NO_2_), may contribute significantly to higher rates of infections and mortality for respiratory and cardiovascular system diseases [1,9,10]. On the basis of epidemiological and experimental studies including smog creation mechanisms, the damage of the respiratory system by PM 2.5 dust was confirmed [10]. Huang et al. postulate that the reduction in visibility due to the high air pollution with co-existing high air humidity had a drastic impact on increasing the incidence of cardiovascular disease and thus increasing mortality among the Chinese population [11]. Katanoda et al. also confirmed the direct relationship between long-term exposure to polluted air and respiratory disease occurrence, including lung cancer [12]. In other studies, it was demonstrated that air pollution can also affect the heart rate and its rhythm, and suggested that this may be one of the reasons for the increased number of strokes and cardiac problems during the days of increased air pollution [13].

For this reason, intensified efforts should be undertaken to eliminate the sources of smog emission, and until the acceptable levels of smog concentrations are achieved, it is advisable to use respiratory protection in the simplest form, which is filtering half masks that meet the essential safety and health requirements contained in the Regulation (EU) 2016/425 of the European Parliament and of the Council of 9 March 2016 [14].

Increasing people’s awareness of health and environmental protection has resulted in an increased popularity of filtering half masks, which protect the respiratory system mostly against aerosols (dust, smoke and mist) in everyday life. As is well known, due to their basic configuration, these masks do not contain the additional layer of sorbent that can protect the users against both solid state particles, such as PM 2.5 or PM 10, and harmful chemical vapors [15,16]. In order to increase protection against volatile organic compounds (VOCs), it is necessary to use protective equipment with absorbers.

Currently, there are no solutions on the market and in scientific journals that would provide the user with a high level of health and life protection against harmful chemical vapors in smog. The half mask protection efficiency depends primarily on the type of used non-woven fabric. To ensure protection against aerosol particles and chemical vapors simultaneously, personal protection masks should be made of a material or its composites that will ensure effective filtration, as well as the absorption of chemical contamination.

Brochocka et al. proposed a non-woven fabric composite containing the inorganic mesoporous silica material type MCM-41, which creates an absorbent layer that extends the breakthrough time against ammonia vapors and composites containing the molecular sieves (SM-zeolite ZSM-5) and in turn extends the breakthrough time for volatile organic solvents, such as acetone and cyclohexane [17].

Carbon-based sorbents, due to their unique physical and chemical properties, have been used for many years as a sorption material in special respiratory protective equipment, for purifying the air of various types of air pollution in gaseous form [18]. The large specific surface area, high porosity, and various shapes and sizes of pores in the entire volume improves the contact between the particles of the adsorbed hazardous substance and active centers spread across the sorbent surface. The size and shape of the pores additionally ensures the efficient transport of the adsorbed substance inside the carbon-based materials. There is a straight relationship between the specific surface area (SSA) of the carbon-based materials, porosity, and size. With the increase in porosity, SSA also increases [19,20,21].

Carbon sorbents are mainly made from natural organic materials, such as wood, peat, lignite, coal, and coconut shells, or various waste materials, such as biomass or plastics [22,23]. In many cases, the characteristics of the microporous structure and the chemical nature of the internal surface are dependent on the raw material from which they are made. Additionally, this also has indirect impact on the adsorption–desorption properties, as well as the kinetics and dynamics of the adsorption of specific adsorbates. A method of the production of composites with filtering and absorbing properties consisting of introducing pulverized activated carbon into the structure of non-woven fabric with the use of a compressed air stream was presented more than a decade ago [24]. A similar issue, concerning the filtering and absorbing properties of the fibrous structures made from effective polypropylene non-woven fabrics formed with the melt-blown technique and non-woven fabric composite with activated carbon, was presented four years later [25].

Recently, Brochocka et al. demonstrated the possibility of introducing additives into the structure of polymer fibers and the production of homogeneous layers of non-woven fabric using melt-blown technology [26]. The use of polymeric non-woven fabrics containing activated carbon in respiratory protective devices results in a significant odor reduction without impeding their functional properties. Authors showed that activated carbons protect against organic compounds (cyclohexane and acetone). On the other hand, additives of mesoporous silica increase the absorption of inorganic compounds, such as ammonia vapors. Therefore, it is important to introduce modifiers of different quantitative and qualitative composition into the structure of polymer fibers due to the specific purpose of these materials.

The permissible concentration values of hazardous smog components in atmospheric air (expressed in µg/m^3^) varies from the maximum to the minimum admissible concentrations in the work environment (MACs). For example, in regard to PM 2.5 dust, this concentration is 12 times lower than MAC [27]. For benzene, it is even 300 times lower than MAC [27]. Such large differences are a consequence of the following aspects taken into account during the determining process of the respective values:

1. Exposure time (MAC—8 h working day vs. daily/yearly breathable air) [28];
2. Impact not only on healthy people (admitted to work), but also on the most vulnerable groups of society (children, the elderly, and people suffering for respiratory and circulatory system diseases or allergies).

On the other hand, exposure to chemical compounds and dust in the air of the working environment occurs when the MAC value is exceeded. MACs are determined for healthy people (admitted to work) and refer to an 8 h working day, average weekly working time and 40 years of work. These values are specified in mg/m^3^ in the Regulation of the Minister of Labor and Social Policy of 12 June 2018 [29].

The exposure of the atmospheric to air pollutants—smog—requires action to protect the general public. People who are exposed to smog for a longer time or whose health condition, due to respiratory, circulatory, or allergic diseases, indicates the need for special protection, should use filtering half masks with CE marking, confirming compliance with the requirements for protection against aerosols (dusts, fumes, and mists). Unfortunately, filtering half masks with the commercial name of “anti-smog”, which do not have the CE marking, have appeared on the market, and do not meet any requirements for protection against dust or, even more so, against chemicals. The use of innovative materials with filtering–absorbing properties in half masks will make it possible to protect the respiratory system against all air pollutants present in smog (as well aerosols and chemical gases and vapors).

All currently available solutions for filtering half masks are related to the protection of the respiratory system, primarily against aerosols (dust, smoke, and mists). There is no mention of a non-woven fabric produced with the melt-blown technique containing a layer with carbon sorbent, or information that the half masks can also protect against vapors and gases present in smog. The fabrication of a filtering–absorbing material that protects against aerosols, including PM 2.5–10 dusts and chemical substances, requires the development of a method of introducing the modifier into the structure of polymer fibers. The innovative idea of combining carbon sorbents with non-woven fabric during the melt-blown technique presented in this work showed the possibility of the potential use of these composites in fully protective antismog masks.

## 2. Methods

### 2.1. Textural Studies

The textural parameters of the carbon sorbents were studied using an AutosorbiQ analyzer (Quantachrome Instruments, Boynton Beach, FL, USA), which measures sorption on the surface of solids by the static volumetric method. During the measurements, a set amount of adsorbate was introduced into a cell containing the adsorbent. The cell temperature was maintained at a constant level of −196.15 °C. The samples were previously dehydrated at 120 °C for 12 h.

### 2.2. Microscopic Evaluation of Structural Properties

The evaluation of the morphological properties of the carbon sorbent and fabricated composite materials were carried out with a HITACHI SU8010 high-resolution electron microscope (SEM, Hitachi High-Technology Corporation, Tokyo, Japan). The evaluation concerned the uniformity of distribution of carbon sorbent particles and their attachment to the polymer structure.

### 2.3. Protection and Utility Performance Tests

Analysis of the breakthrough time was intended to determine the time of protection performance of the filtering–adsorbing non-woven fabrics. The protective properties of the carbon sorbents were maintained until the time when the sorption bed was permeated by noxious chemical substances. One of the most important parameters is the protection time period when the composite fulfills its absorbing role. Its value depends on the available sorbent bed capacity, but also on the volume and air flow rate corresponding to the human one-minute lung ventilation rate, concentration of a given substance in the air, and combined presence of several substances in the air, as well as the air temperature and humidity.

Respiratory protective devices made of filtering and absorbing materials are intended not only to ensure protection against dust and other solid particles, but also provide adequate protection against volatile chemical compounds. The test gases were cyclohexane, toluene, and sulfur dioxide, and the permissible concentration values for these substances were 81, 24.6, and 0.46 ppm, respectively (during the test, the permissible variation in the gas concentration should not have exceeded ± 5 ppm versus the initial value), with a relative humidity of 70 ± 5% and a temperature of 21 ± 1 °C. Tests were conducted with a volume flow rate of 30 L/min (according to the test standard EN 14387:2004 + A1:2008) [18].

### 2.4. Paraffin Oil Mist Aerosol Penetration

Paraffin oil mist aerosol penetration was measured in accordance with the European standards concerning the test methods and requirements for filtering half masks EN 149:2001 + A1:2009 [30] and EN 13274-7:2008 [31]. The aerosol was produced using a Lorenz AGW-F/BIA generator (LORENTZ, Lindau, Germany) and passed at a linear flow rate of 95 L/min through a non-woven fabric sample placed in a FH 143/149 pneumatic holder with a diameter of 113 mm. The aerosol concentration upstream and downstream of the non-woven fabric sample was measured using a Lorenz AP2E laser photometer (LORENTZ, Lindau, Germany) after 3 min of the experiment (in the initial phase of filtration) and expressed as a percentage.

### 2.5. Air Flow Resistance

Airflow resistance was determined according to the European standards concerning test methods and requirements for filtering half masks EN 149:2001 + A1:2009 [30] and EN 13274-3:2008 [32]. In exhalation phase tests, air was passed through non-woven fabric samples at a constant volumetric flow rate, and the downstream pressure differential was measured with respect to the atmospheric pressure. The airflow rate was 95 L/min in accordance with the abovementioned European Standard.

## 3. Materials

Isotactic polypropylene (PP) granulate Borealis HL 508J (NEXEO Solutions Poland Sp. z o.o., Warsaw, Poland) was used as a polymer matrix to produce the filtration non-woven fabric material, which is characterized by the following parameters: melting point: 156 °C–160 °C; melt flow index (MFI): 800 g/10 min; density: 50.3 g/cm^2^.

The filtering–absorbing materials were prepared by the melt-blown technique. In this method, non-woven fabrics were prepared by blowing the melted PP into elementary fibers of various diameters (from 100 nm to 5 µm) and lengths, where carbon sorbent was introduced directly through the fiber-forming head into the stream of elementary polymer fibers. The mixture of PP fibers (20 g/m^2^) with the addition of carbon sorbent in the amount of 30 g/m^2^ was collected and subjected to an electrostatic activation process at 25 kV, creating a filtering–absorbing material with a mass in the range of 50 g/m^2^. In order to increase the amount of carbon sorbent in the fabric, single layers of non-woven fabrics were superimposed to achieve a total amount of carbon of 60, 90, 120, 150, and 210 g, increasing the surface mass of the material to 100, 150, 200, 250, and 300 g/m^2^, respectively. It should be mentioned that, regardless of the number of “pure” non-woven fabric layers, the breakthrough time for gas vapors is comparable and negligible.

Two types of commercial carbon sorbent were used in the production of the filtering–absorbing materials:

- AG Pleisch, 30180 PL MC 12 × 20 CBRN (manufacturer: PLEISCH, Bäretswil, Switzerland);
- SAR (supplier: Zakład Doświadczalny Produkcji Węgli Aktywnych, Mrozy, Poland).

### 3.1. Preparation of Carbon Sorbents

The carbon sorbents available on the market have varying dimensional distributions of particle sizes, mainly above 300 μm, whereas the melt-blown filtration non-woven fabrics have a fiber diameter of approximately 1 μm [33]. In order to permanently attach sorbent particles to the elementary PP fibers, the size of the sorbent particles should be comparable to the fiber diameter [34]. This required the proper preparation of the sorbents to obtain the appropriate granulation and specific dimensional distribution of the grains. To obtain these desired properties, the carbon sorbent was pulverized in a planetary ball mill (Retsch GmbH, Haan, Germany).

**Figure 1:**
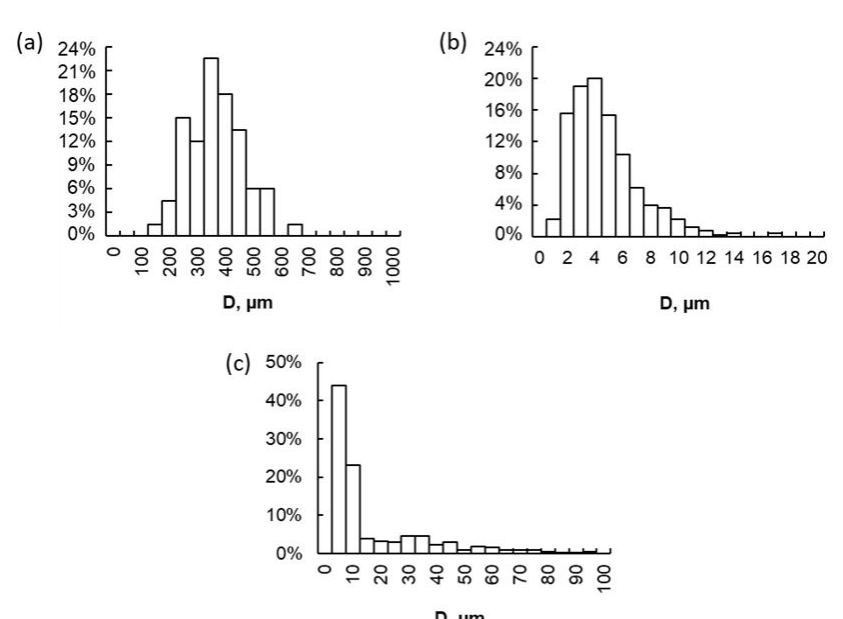
Distribution of particle size of AG Pleisch carbon sorbent before grinding (**a**) and after four hours of grinding in a planetary ball mill, (**b**) and of SAR carbon sorbent after four hours of grinding (**c**).

In order to determine the size of the carbon sorbent after the grinding process, a stereological analysis was carried out. The AG Pleisch carbon sorbent was used as a reference sample. To optimize the specific dimensional distribution and select the most suitable sorbent grinding degree, the grinding process was carried out for 5, 20, 40, 60, 120, 240, and 360 min. Figure 1 shows the distribution of the average particle size before the grinding for AG Pleisch and after 240 min of gridding process for AG Pleisch and SAR. The average diameter of AG Pleisch before grinding was 342 µm, whereas grinding for 240 min led to an average size of the AG Pleisch particles equal to 4 µm (Figure 1b). In the case of the SAR carbon sorbent, which is supplied in a much larger granulate size, the particle size distribution was determined only after the 240 min grinding process (Figure 1c). The size of particles can be divided into two fractions. The main fraction of ~66% possesses a narrow size distribution, below 10 µm, whereas the other 36% particles have a wide size distribution, from 10 to 80 µm. The 240 min grinding time is optimal in terms of obtaining a homogeneous distribution of the sorbent particle size. Extending the time causes the excessive fragmentation of the carbon-based sorbent, which causes them to stick together and the drastic deterioration of the assembly properties.

**Figure 2:**
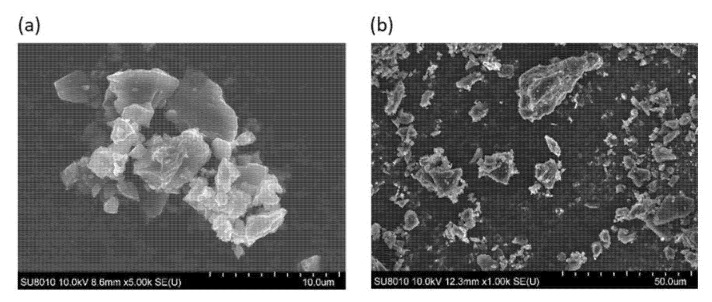
Images of pulverized carbon sorbent after 240 min: (**a**) AG Pleisch (×5000 magnification); (**b**) SAR (×1000 magnification).

Figure 2 shows SEM images, which demonstrate the structure of the investigated pulverized carbon sorbent. In the case of AG Pleisch, it is characterized by the large fragmentation (around 4 µm) of the material, which forms larger agglomerates with dimensions of above 25 µm (Figure 2a). SAR, on the other hand, possesses a wide distribution of different particle sizes. However, SEM images (Figure 2b) clearly demonstrate that a significant fraction of the particle sizes are equal to 10 µm.

Visible differences in the morphologies between AG Pleisch and SAR carbon sorbents can be assigned to the different hardnesses of those materials. AG Pleisch, due to its lower hardness, forms larger, more dense agglomerates with a diameter of >15 µm. On the other hand, the SAR carbon sorbent possesses greater material hardness, which causes a wide particle size distribution without visible agglomerates.

### 3.2. Textural Parameters of Carbon Sorbents

To determine the dependence of grinding time on textural properties such micropore volume (t-plot), micropore diameter (DR), and specific surface area (SSA), the static volumetric method was applied. The results of the analysis are presented in Table 1.

It was found that unprocessed SAR activated carbon possesses a much lower specific surface area in comparison to AG Pleisch which are 934 and 1164 m^2^/g, respectively. Differences in the SSA value may be directly related to the production methods of these materials. Additionally, it was found that the grinding process has a negative impact on SSA parameters, and extending the grinding time reduced the active surface area of the materials. This phenomenon occurred for both materials and is related to the process of sample crushing, during which the pores in the material are clogged and closed. In the case of AG Pleisch, 240 min of gridding reduced the SSA to 1093 m^2^/g, and for SAR, to 852 m^2^/g.

The micropore volume (t-plot) exhibited a similar relation to the SSA parameters, and its value did not change significantly during the grinding process. In the case of AG Pleisch, the visible fluctuation in value was within the error limit; however, for the SAR carbon sorbent, significant differences were observed. The changed value from 0.410 to 0.381 cm^3^/g suggests that these carbon types should possess a higher capacity of chemical compounds captured by way of physical adsorption. Due to the fact that the grinding process did not adversely affect the textural properties of the carbon sorbent, a suitable degree of grinding should be selected for the chosen raw material fabrication process.

## 4. Results and Discussion

### 4.1. Microscopic Morphological Structure Evaluation

**Figure 3:**
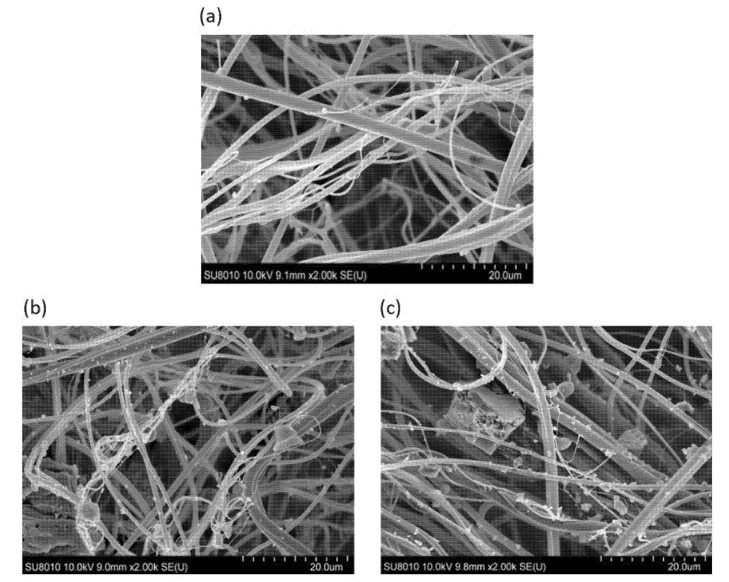
SEM images of the polypropylene filtration non-woven fabric without sorbent: (**a**) PP non-woven fabric containing; (**b**) 30 g of AG Pleisch carbon sorbent after 240 min of pulverizing and PP non-woven fabric containing; (**c**) 30 g of SAR carbon sorbent after 240 min of pulverizing.

Figure 3 shows the images from the scanning electron microscope (SEM) of the morphological structures of bare non-woven fabrics and non-woven fabrics with carbon sorbents: AG Pleisch and SAR.

Based on the analysis of the SEM images, it was found that all non-woven fabrics were characterized by randomly arranged primary fibers with diameters in the range of 1–4 µm (Figure 3a). Fibers formed a compact and tangled network with openings of no more than 20 µm.

The introduction of additives to the melt-blown process of PP created a non-woven fabric with carbon sorbents evenly distributed throughout the entire volume. Figure 3b,c demonstrate SEM images of the obtained non-woven fabrics with carbon additives. It is shown that individual particles with sizes comparable to the diameters of individual fibers were attached directly to them, while the larger particles were located in voids between the tangled polymer networks. Tight PP non-woven fabric ensured the stability of the position of the carbon sorbents. Additionally, the size distribution of the activated carbon sorbent particles was in agreement with the measurements of pure carbon sorbent size. In the case of AG Pleisch (Figure 3b), it was easy to distinguish a large number of carbon particles with sizes equal to or less than 1 µm, while at the same time, much larger particles, as well as agglomerates, with diameters of greater than 15 µm were observed independently of the amount of the used carbon.

SAR (Figure 3c) particles have a different size distribution. It was easy to distinguish that a large amount of particles with dimensions in the range of 3–5 µm (much larger than those observed for AG Pleisch) were attached to the fibers. Additionally, fractions of particles of larger than 25 µm were visible.

### 4.2. Sorption Properties—Breakthrough Time

**Figure 4:**
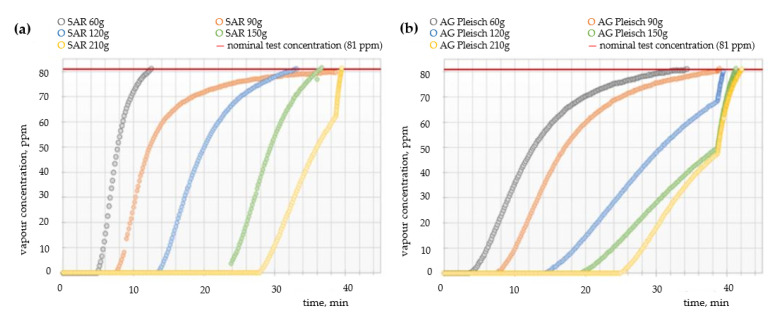
Breakthrough time curves of cyclohexane vapors for filtration fabrics with different contents of (**a**) SAR and (**b**) AG Pleisch sorbents.

**Figure 5:**
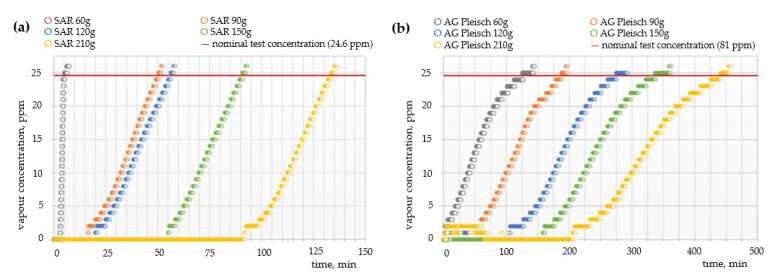
Breakthrough time curves of toluene vapors for filtration fabrics with different contents of (**a**) SAR and (**b**) AG Pleisch sorbents.

**Figure 6:**
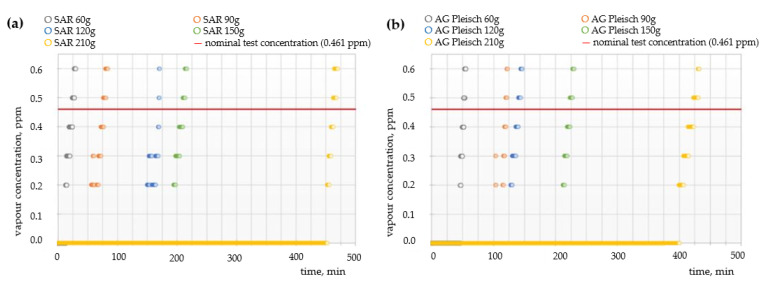
Breakthrough time curves of sulfur oxide vapors for filtration fabrics with different contents of (**a**) SAR and (**b**) AG Pleisch sorbents.

Figure 4, Figure 5 and Figure 6 show the breakthrough time curves of cyclohexane, toluene, and sulfur dioxide vapors for filtration non-woven fabrics with carbon sorbents of varying quantitative and qualitative compositions.

Cyclohexane was chosen to perform the experiments due to the fact that it is the representative of protection group A (organic gases) named in the test standard EN 14387:2004 + A1:2008 [18]. Cyclohexane is a good representative of this group of substances because it is non-polar and non-reactive and, therefore, should be bound to the carbon surface mainly by physical adsorption. In addition, it is one of the compounds that are used in large amounts in industrial processes; hence, it is often found in the work environment. Results presented in Figure 4 demonstrate that the saturation process is highly dependent on the amount of carbon sorbents in the composite material. In the case of the SAR sorbent, increasing the amount of carbon additives from 60 to 210 g simultaneously increases the breakthrough time by almost four times, from 12 up to 40 min, respectively. As expected, AG Pleisch exhibited similar properties to the SAR sorbent; however, the extension of the breakthrough time between the minimum and maximum amount of sorbents was only 8 min, with full saturation achieved after 42 min for the composite with 210 g of carbon sorbent. Differences in curve shapes and reaction times between SAR and AG Pleisch are directly attributed to the textural parameters of these two materials and interactions between the cyclohexane and carbon sorbents.

In the next step, the breakthrough time curves of toluene vapors were determined (Figure 5). Toluene is an organic compound classified as toxic and dangerous, often present in urban smog and in households as a component of paints and plastics, posing an inhalation hazard. Again, it was found that the filtration efficiency of the AG Pleisch activated carbon provided considerably better protection against toluene vapor than that of SAR. A proportional increase in the time of protective action with the quantity of applied carbon sorbent was observed, both for the SAR and AG Pleisch activated carbons. For the SAR carbon sorbent, the breakthrough time ranged from 7 up to 130 min. The AG Pleisch sorbent may provide much better protection against toluene vapor in comparison to SAR. The addition of 60 g of this carbon sorbent provides protection for more than 120 min, while increasing the amount of additives by up to 210 g of carbon sorbent provides 450 min of protection. This is almost a 3.5 times longer performance than the SAR filled filter with the highest amount of sorbent.

One of the main components of smog is an inorganic chemical compound, sulfur dioxide (SO_2_). Due to the limitation of the used equipment, it was complicated to obtain a stable flow of gas medium with low concentrations of SO_2_. Therefore, the test was performed under constant gas flow with 12 ppm of SO_2_—which was the lowest stable flow with a well-defined concentration of the tested gas. Figure 6 shows the results of the breakthrough time tests. Both tested carbon sorbents possess similar absorption properties for sulfur dioxide vapors; however, the SAR sorbent exhibited a slightly longer breakthrough time in comparison to AG Pleisch. The highest amount of SAR sorbent (210 g) provided protection against SO_2_ vapors for 470 min, while 210 g of AG Pleisch provided protection for 425 min. Moreover, the analysis of the breakthrough curves demonstrated that there was no linear relation between the breakthrough time and amount of carbon sorbent quantity. The increase of both sorbents amount from 150 g to 210 g extend the breakthrough time twice.

The results of the analyses are in agreement with the theory posed in the literature, which states that the breakthrough time of activated carbon absorbers under ambient conditions is proportional to the absorbed substance concentration [26]. Based on the performed measurements, it was clearly visible that increasing the amount of activated carbon sorbent in polypropylene non-woven fabrics extended the breakthrough time with almost linear trends. Nevertheless, studies have shown the differences between tested absorbers. The AG Pleisch carbon sorbent exhibited generally higher absorption properties in comparison to SAR against the toluene and cyclohexane vapors, where SAR revealed slightly longer protective times against sulfur dioxide vapors. The better absorption properties of AG Pleisch are directly related to its specific surface area, which is 20% higher than that of the SAR carbon sorbent. An interesting observation, in turn, is the sharp shape of the breakthrough time curves for all vapors (or gases), especially from the beginning of the test. At low concentrations, certain regions in the material became completely saturated, subsequently allowing the vapor molecules to travel through the regions still holding some unspent capacity. Thus, the breakthrough curve was sharp as penetration started but became elongated as certain regions in the material slowly became saturated.

### 4.3. Protective and Functional Parameters

In order to investigate the protective and functional parameters of the manufactured non-woven fabrics, aerosol penetration of paraffin oil mist tests were performed. For this purpose, three types of non-woven fabrics were tested, and the results of the analysis are presented in Table 2.

In the case of non-charged bare non-woven fabric, the aerosol penetration of paraffin oil mist coefficient was approximately 6%. The introduction of an electrostatic charge by corona discharge in the non-woven fabric fabrication process resulted in a significant improvement in the filtration properties and a reduction in the penetration down to 2% (3 times less). The introduction of 210 g of the AG Pleisch carbon sorbent into the structure of the non-woven fabric resulted in a further decrease in penetration index to 1% in comparison to bare non-woven fabrics.

In addition, measurements of the air flow resistance showed that PP non-woven fabric is characterized by a flow resistance of 159 Pa. Corona discharged non-woven fabric possesses a slightly lower flow resistance of 137 Pa, which may be caused by a difference in the weight between the variants of non-woven fabrics, especially since they are three-dimensional structures with disordered distributions of fibers. The produced composite with 210 g of AG Pleisch was characterized by the lowest air flow resistance of 119 Pa. The introduction of carbon sorbent particles with sizes of approximately 4 µm to the structure of the filter material did not deteriorate the filtration and protective parameters. This experiment proved that the addition of the activated carbon sorbent does not affect the comfort of use, and this type of composite can be used in smog protection masks.

## 5. Conclusions

This study presents innovative non-woven fabrics with activated carbon additives fabricated by one technological process. The modification of the non-woven fabric structure by the introduction of carbon additives directly in the fiber forming process may be successfully used for the production of filtering materials for air-purifying half masks and increase their versatility by adding absorption properties for chemical air pollution.

Taking into account the extension of the protective time depending on the amount of carbon sorbent, the SAR sorbent was found to be a better variant. On the other hand, the AG Pleisch-type sorbent was characterized by longer protection times with smaller amounts in the filtering material. In the case of the breakthrough time of cyclohexane vapor for the absorbent material, it provided effective protection for about 25 min. As a result of the conducted research, it was observed that it provided much better protection against the other two test substances: toluene and sulfur dioxide, for which the effective production of action was over 2 and 6 h, respectively. It was revealed that, compared to commercially available products [35], the presented absorbing material showed much better protective properties. Moreover, the filtering material containing a smaller amount of carbon sorbent revealed lower air flow resistance, which would have a great impact on the comfort of use, if such non-woven fabric was applied in smog protective half masks. The studies presented in this paper studies will be continued towards the optimization of the introduction of carbon sorbent particles to filtering materials to prevent the agglomeration of particles and optimize the amount of used carbon sorbents.

## Figures and Tables

**Table 1:** Textural and adsorption properties of activated carbon samples.

Sample	Micropore Volume (t-Plot), cm^3^/g	Micropore Diameter (DR), nm	Specific Surface Area (BET), m^2^/g
AG Pleisch	0.452	1.38	1164
AG Pleisch pulverized 60 min	0.434	1.34	1137
AG Pleisch pulverized 120 min	0.389	1.14	1015
AG Pleisch pulverized 240 min	0.418	1.40	1093
AG Pleisch pulverized 360 min	0.405	-	1021
SAR	0.410	1.54	934
SAR pulverized 240 min	0.352	-	852

**Table 2:** Summary of basic protective and operational parameters for filtering and filtering–absorbing materials for use in smog half masks.

Parameter	PP	Electrostatically Charged PP	PP + AG Pleisch
Penetration against oil mist aerosol, %	6.6	1.9	1.1
Air flow resistance, Pa	159	137	119

## Data Availability

The data presented in this study are available on request from the corresponding author.
